# Supplementary figures and images for: Juvenile Hormone Involved in the Defensive Behaviors of Soldiers in Termite Reticulitermes aculabialis
Source: Insects. 2024 Feb 14;15(2):130. doi: 10.3390/insects15020130 (PMC10889337; doi:10.3390/insects15020130)

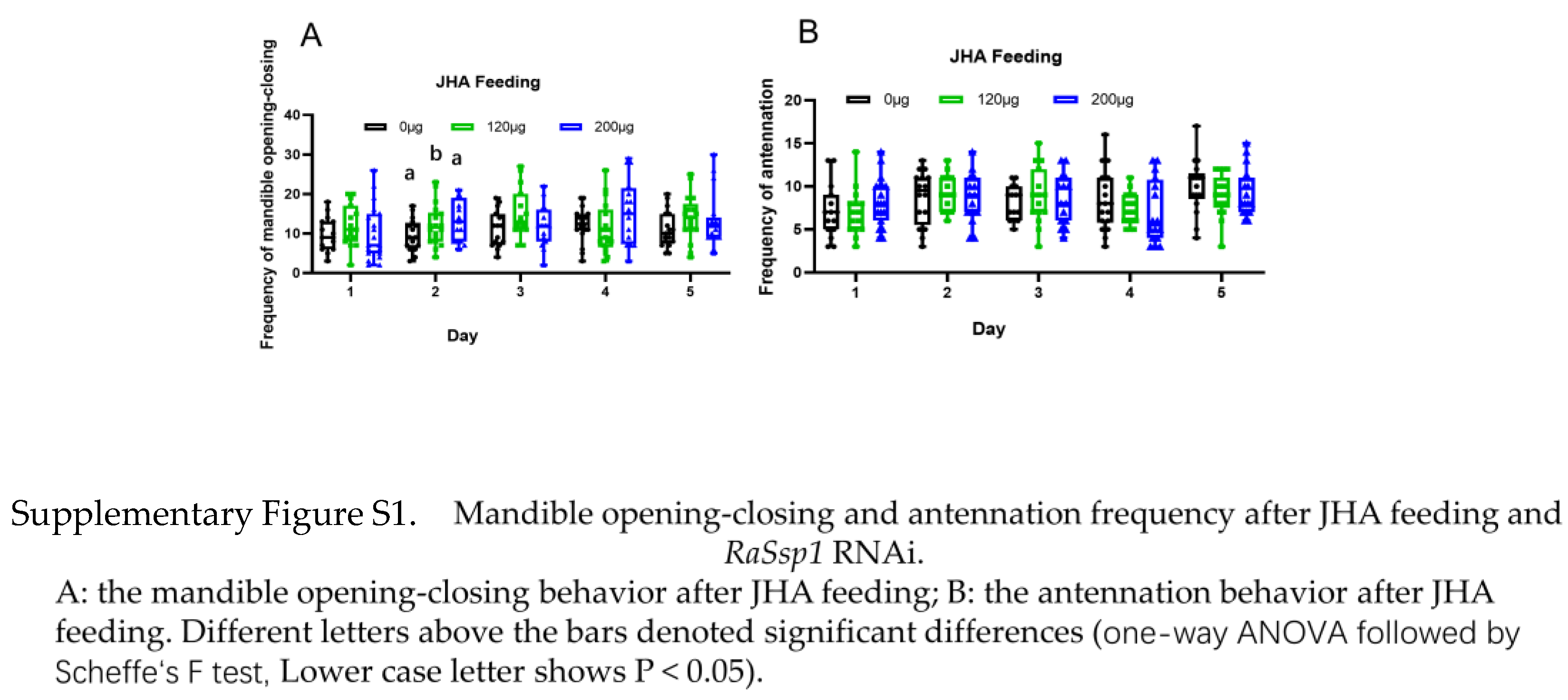

Supplement: Supplementary file 1 [file insects-15-00130-s001.zip › insects-2815337-Figure S1.tif]
